# Cardiac fibrogenesis: an immuno-metabolic perspective

**DOI:** 10.3389/fphys.2024.1336551

**Published:** 2024-03-21

**Authors:** Md Monirul Hoque, Joy Olaoluwa Gbadegoye, Fasilat Oluwakemi Hassan, Amr Raafat, Djamel Lebeche

**Affiliations:** ^1^ Departments of Physiology, The University of Tennessee Health Science Center, Memphis, TN, United States; ^2^ College of Graduate Health Sciences, The University of Tennessee Health Science Center, Memphis, TN, United States; ^3^ Medicine-Cardiology, College of Medicine, The University of Tennessee Health Science Center, Memphis, TN, United States; ^4^ Pharmaceutical Sciences, College of Pharmacy, The University of Tennessee Health Science Center, Memphis, TN, United States

**Keywords:** cardiac fibrosis, immune cells, immunometabolism, cardiometabolism, inflammation, metabolic reprogramming

## Abstract

Cardiac fibrosis is a major and complex pathophysiological process that ultimately culminates in cardiac dysfunction and heart failure. This phenomenon includes not only the replacement of the damaged tissue by a fibrotic scar produced by activated fibroblasts/myofibroblasts but also a spatiotemporal alteration of the structural, biochemical, and biomechanical parameters in the ventricular wall, eliciting a reactive remodeling process. Though mechanical stress, post-infarct homeostatic imbalances, and neurohormonal activation are classically attributed to cardiac fibrosis, emerging evidence that supports the roles of immune system modulation, inflammation, and metabolic dysregulation in the initiation and progression of cardiac fibrogenesis has been reported. Adaptive changes, immune cell phenoconversions, and metabolic shifts in the cardiac nonmyocyte population provide initial protection, but persistent altered metabolic demand eventually contributes to adverse remodeling of the heart. Altered energy metabolism, mitochondrial dysfunction, various immune cells, immune mediators, and cross-talks between the immune cells and cardiomyocytes play crucial roles in orchestrating the transdifferentiation of fibroblasts and ensuing fibrotic remodeling of the heart. Manipulation of the metabolic plasticity, fibroblast–myofibroblast transition, and modulation of the immune response may hold promise for favorably modulating the fibrotic response following different cardiovascular pathological processes. Although the immunologic and metabolic perspectives of fibrosis in the heart are being reported in the literature, they lack a comprehensive sketch bridging these two arenas and illustrating the synchrony between them. This review aims to provide a comprehensive overview of the intricate relationship between different cardiac immune cells and metabolic pathways as well as summarizes the current understanding of the involvement of immune–metabolic pathways in cardiac fibrosis and attempts to identify some of the previously unaddressed questions that require further investigation. Moreover, the potential therapeutic strategies and emerging pharmacological interventions, including immune and metabolic modulators, that show promise in preventing or attenuating cardiac fibrosis and restoring cardiac function will be discussed.

## 1 Introduction

Myocardial fibrosis is a common pathophysiologic companion of many different myocardial conditions, where the cardiac interstitium expands through the deposition of extracellular matrix (ECM) proteins (Frangogiannis, 2021)**.** Unlike other organs, the adult mammalian heart has limited regenerative potential. In response to ischemic insults, systemic diseases, or any other harmful stimulus to the circulatory system or the heart itself, the damaged cardiomyocytes are replaced by a fibrotic scar (Gyöngyösi et al., 2017)**.** Though this event is crucial for the preservation of ventricular rupture (Travers et al., 2016)**,** over time, excessive and continuous ECM deposition leads to irreversible ventricular remodeling and distorted organ geometry and significantly impairs the function of the heart (Li et al., 2018; Maruyama and Imanaka-Yoshida, 2022; Majid et al., 2023)**.**

**FIGURE 1:**
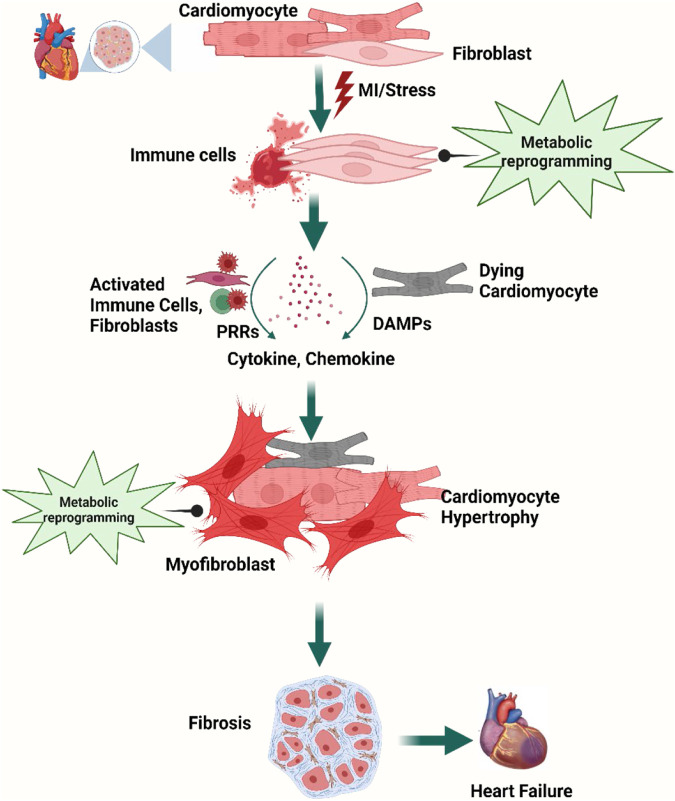
Overview of cellular steps leading to cardiac fibrosis. Following injury, cardiac fibroblasts are activated and transdifferentiated into myofibroblasts to replace dying cardiomyocytes. Dying myocytes release damage-associated molecular patterns (DAMPs) which activate immune cells and trigger downstream signaling cascades of pro-inflammatory cytokines and chemokines. A plethora of cytokines and chemokines in addition to myofibroblasts undergo metabolic alterations that drive the development and progression of myocardial fibrosis and heart failure. Figure created using BioRender.

A wide repertoire of cell populations, including cardiomyocytes, fibroblasts, endothelial cells, smooth muscle cells, and pericytes; different types of immune cells (myeloid and lymphoid); adipocytes; mesothelial cells; and neuronal cells synchronously maintain cardiac function (Meilhac and Buckingham, 2018; Litviňuková et al., 2020; Tucker et al., 2020; Marín-Sedeño et al., 2021)**.** Sustenance of cardiac homeostasis depends on the integrity of individual cells and the cellular interactions mediated by juxtacrine, paracrine, and endocrine signals. Any pathogenic stimuli disrupting the cardiac microenvironment, metabolic demand, hemodynamic stability, or these crosstalk networks strain the cardiomyocytes and lead to counter-responses (Pogontke et al., 2019; Marín-Sedeño et al., 2021; Liu et al., 2023) and initiation of an inflammatory cascade (Hara and Tallquist, 2023)**.** Activation of the resident immune cells as well as recruitment of innate and adaptive immune cells attempt to adapt to the insult. However, the activation of pattern recognition receptors (PRRs) by damage-associated molecular patterns (DAMPs) or pathogen-associated molecular patterns (PAMPs) initiates downstream signaling cascades that might upregulate the expression of genes encoding pro-inflammatory cytokines and chemokines (Mann, 2011; Adamo et al., 2020a; Silvis et al., 2020)**.** Persistent low-grade inflammation activates tissue-resident macrophages and mast cells to recruit and activate B and T cells, which trigger plasma protein infiltration. The temporal imbalance between the activity of matrix metalloproteinases (MMPs) and tissue inhibitor of MMPs (TIMPs) with a collateral increase in transforming growth factor-β (TGF-β) signaling and NLR family pyrin domain-containing 3 (NLRP3) inflammasome formation leads to aberrant fibrotic deposition and left ventricular (LV) remodeling (Mezzaroma et al., 2011; Zhang et al., 2011; Adamo et al., 2020a)**.** Changes in cardiac performances resulting from altered intercellular signaling, loss of cell activity, or cell death can then result in further changes in cell-to-cell communication (Buckingham et al., 2005; Marín-Sedeño et al., 2021) (Figure 1).

Along with immune activation, metabolic reprogramming, supply–demand mismatch, and disruption of the equilibrium in local metabolites in the affected cardiac tissue contribute to progression of cardiac fibrosis. Metabolic reprogramming allows the cells to adopt dynamic changes in cellular metabolic pathways and biological functions in response to various stimuli or environmental conditions, for example, cellular metabolism switches from oxidative metabolism (i.e., oxidative phosphorylation, OXPHOS) to the more oxygen-sparing carbohydrate metabolism (i.e., glycolysis) and utilization of glutamine and fatty acid increases to meet the energy and biosynthetic demands during acute and chronic cardiac stress (Sun et al., 2020a; Sabogal-Guáqueta et al., 2023; Ritterhoff and Tian, 2017; Rosano and Vitale, 2018; Yoganathan et al., 2023; Razeghi et al., 2001; Akki et al., 2008; Aubert et al., 2016; Bedi et al., 2016; Tran and Wang, 2019; Lin et al., 2023)**.** With progressive low-grade inflammation in the background of cardiac insult, cytokines and nutrient metabolites activate inflammatory programs through shared pathways, and resident and recruited immune cells undergo metabolic shifts on their own as well during fibrotic events (Sun et al., 2020a; Sabogal-Guáqueta et al., 2023). The energy demand and metabolic intermediates for immune cell activation and differentiation are dependent more on glycolysis than on the tricarboxylic acid (TCA) cycle and OXPHOS (Srivastava et al., 2018; Cho et al., 2020; Farah et al., 2021; Wenzl et al., 2021)**.** Moreover, the fibroblasts adopt a metabolic phenotype using glycolysis and glutamine-derived α-ketoglutarate to adapt to the altered microenvironment (Kawaguchi et al., 2011; Mouton and Hall, 2020; Farah et al., 2021; Francisco et al., 2021).

Immunometabolism is still a burgeoning field, and much of the existing knowledge on cardiac immune cell metabolism is based on myocardial infarction (MI) models (Mouton and Hall, 2020)**.** In the past few years, technological advances and highly sensitive metabolomics approaches have redefined the inextricable relationships between immune activation, molecular signaling, and metabolism and discovered their association to immune cell functions in the course of the disease. However, there are knowledge gaps in the complete sketch of immunometabolism from the perspective of cardiac fibrosis that require further exploration to use immune modulation strategies to prevent cardiac remodeling.

In this review, we provide an update on the current understanding of the involvement of immune and metabolic systems in cardiac fibrosis and the potential of immune–metabolic reprogramming in the management of cardiac fibrosis and restoration of cardiac function.

## 2 Contribution of the immune system to cardiac homeostasis

A large number of innate and adaptive immune cells are found in the heart. Following infiltration of the cardiac tissue at gestation, the immune cells persist in the myocardium and engage in essential housekeeping functions; defend against pathogens, toxic insults, hypoxia, or other injury; and maintain normal cardiac function throughout life. Though the resident and recruited immune cell populations change in different stages of life as well as over the course of injurious stimuli, the major subtypes of inflammatory cells include leukocytes, mononuclear phagocytes, neutrophils, B cells, and T cells. The complex interactions and crosstalk between different subsets of immune cells that either reside or infiltrate the cardiac tissue and the resident cardiac and non-cardiac cells comprising cardiomyocytes, fibroblasts, and endothelial cells maintain the physiological microenvironment in the heart (Pinto et al., 2012; Ramos et al., 2017; Swirski and Nahrendorf, 2018; Marelli-Berg and Aksentijevic, 2019; Steffens et al., 2022).

Architecturally, the heart is heterogeneous, as is the distribution of immune cells: various macrophage subsets are non-uniformly distributed in distinct niches of the heart. Dendritic cells are found abundantly in the cardiac valves and aortic sinus (Choi et al., 2009), whereas the atrioventricular node contains a high concentration of tissue-resident macrophages (Hulsmans et al., 2017). On the other hand, the coronary vasculature is rich in CCR2^-^ (CC chemokine receptor 2) macrophages and fetal monocyte-derived macrophages are concentrated adjacent to the endocardial trabeculae (Leid et al., 2016). These findings suggest that biochemical, neurohormonal, nutritional, or metabolic alterations of different niches of the heart involve different populations of inflammatory cells as principal responders and lead to different pathophysiological courses. Moreover, the organized chambers, vessels, and myocardium are immersed in the serosal fluid within the pericardium, which contains leukocytes, macrophages, and B cells and provides tissue-infiltrating leukocytes during challenges (Butts et al., 2017). The pericardial adipose tissue supplies lymphocytes and coordinates granulopoiesis and the activation of immune cells (Horckmans et al., 2018)**,** whereas white adipose tissue synchronizes the supply and mast cell accumulation in the heart following MI (Ngkelo et al., 2016). We lack a complete picture of the spatiotemporal distribution of different immune cell populations. A summary of the immune cells along with their distribution and role in the heart is enlisted in Table 1.

**TABLE 1:** Role of different immune cells in healthy and ischemic heart.

Type of cell	Distribution in the heart	Role in the healthy heart	Role in ischemic injury	Ref.
Macrophages	Left ventricle, coronary vasculature, endocardial trabeculae, and atrioventricular node	Immunosurveillance of myocardial tissue, maintenance of mitochondrial homeostasis, modulation of electrical activities of cardiomyocytes, growth and remodeling of coronary vessels, stimulation of angiogenesis, and modulation of the local stromal environment	Exert pro-inflammatory signals, followed by reparative cues, mediate post-MI fibrotic response, activate cardiac fibroblasts, promote collagen deposition, and aid in ECM turnover	Heidt et al. (2014), Leid et al. (2016), Hulsmans et al. (2017), Cao et al. (2018), Nicolás-Ávila et al. (2020), Simões et al. (2020), and Revelo et al. (2021)
Monocytes	Patrols the myocardial vasculature, recruited massively on infarction	Mediate immune response, immunosurveillance against pathogens, and toxic insults	Scavenge dead cardiomyocytes and remove debris from the vasculature, Ly-6C (hi) monocytes digest damaged tissue, Ly-6C (lo) monocytes promote healing via myofibroblast accumulation, angiogenesis, and deposition of collagen	Auffray et al. (2007), Nahrendorf et al. (2007), Jung et al. (2013), and Leo et al. (2013)
Neutrophils	Pericardial adipose tissue, recruited upon injuries	Essential for the initiation and resolution of inflammation, primary mediator of the innate host defense	Release ROS and MPO, activate enzymes that degrade the ECM, modulate monocyte/macrophage polarization, LV remodeling, and clear debris in HF	Vasilyev et al. (2005), Ali et al. (2016), and Horckmans et al. (2017)
Lymphocytes	Cardiac interstitium, intravascular space, myocardium, and epicardium harbor B cells, T-cells are found in the serosal fluid, and lymphocytes are recruited following injuries	B cells contribute to immune responses, promote CD4 (+) T-cell polarization via DAMP-mediated activation, and maintain the homeostasis of certain types of NK cells	B cells trigger monocyte mobilization and impair heart function following MI, and CD4 (+) T cells facilitate post-MI wound healing and cardiac remodeling	Hofmann et al. (2012), Zouggari et al. (2013), Adamo et al. (2020b), and Bermea et al. (2022)
Mast cells	Epicardium, white adipose tissue, and mast cell population increase in cardiac volume overload	Recognize DAMPs, release cytokines and mediators, and promote angiogenesis,	Degranulate and release preformed mediators, induce fibroblast activation, and regulate myofibroblast function,	Rakusan et al. (1990), Frangogiannis et al. (1998a), Gailit et al. (2001), Forman et al. (2006), Ngkelo et al. (2016), Ingason et al. (2019)
Dendritic cells	Aortic valves, sinus, and lesser curvature of the aortic arch	Capture disease-related pathogens and proteins and present these to T-cells	Activate lymphocytes by uptake and presentation of myocardial peptides, post-MI LV remodeling, and immunoprotective regulation via modulating monocyte/macrophage homeostasis	Zhang et al. (1993), Naito et al. (2008), Choi et al. (2009), Anzai et al. (2012), and Liu et al. (2016)

**FIGURE 2:**
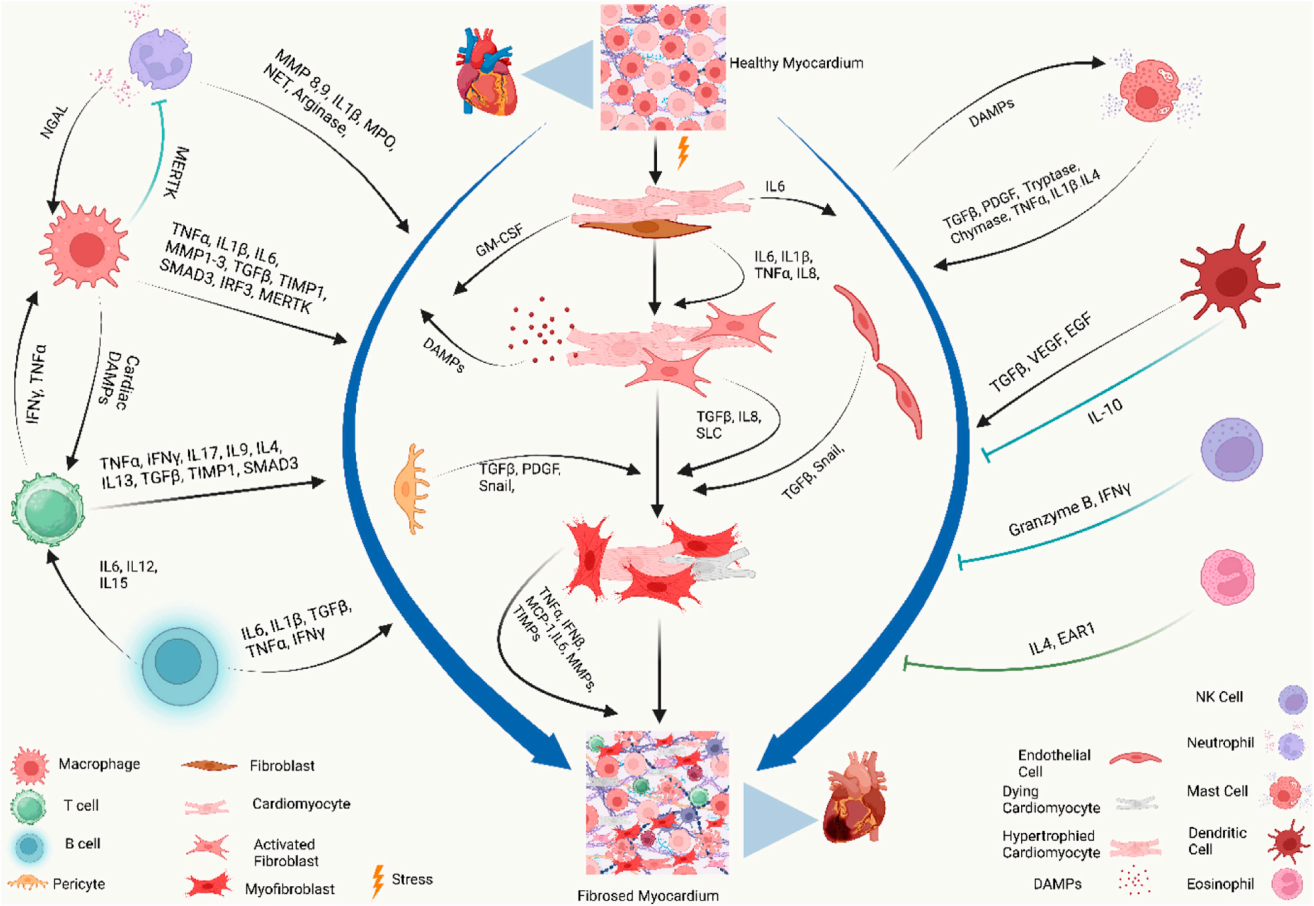
Immune crosstalk between inflammatory cells and cardiac cells during the transformation of the cardiac microenvironment toward fibrosis. The injured myocardium alters the cardiac microenvironment and releases different growth factors and inflammatory mediators, i.e., IL-6, IL-1β, GM-CSF, TNF-α, PDGF, and DAMPs, from injured cardiomyocytes, cardiac fibroblasts, endothelial cells, and pericytes (Gwechenberger et al., 1999; MeléNdez et al., 2010a; Anzai et al., 2017). Upon activation, cardiac fibroblasts further promote the cascades and release MMPs, TIMPs, MCP-1, and TNF-α, which play a role in ECM remodeling and fibrogenesis (Yoshimura et al., 2014; Pluijmert et al., 2021). During these events, the recruited and activated immune cells release another wave of cytokines and chemokines which further promote cardiac remodeling. Recruited monocytes and macrophages trigger inflammatory responses by releasing DAMPs, detect extracellular DNA from dying cells, and release type I interferons (King et al., 2017). Resident macrophages, IL-10 signaling, and MERTK modulate inflammatory cells, promote reparative events, inhibit hyperactivation of fibroblasts, and mitigate fibrosis (DeBerge et al., 2017; Jung et al., 2017; Revelo et al., 2021). Simultaneously, neutrophils, lymphocytes, and mast cells crosstalk with macrophages and fibroblasts to stage the background of ECM turnover and deposition of collagen by releasing inflammatory cytokines. Dendritic cells and NK cells contribute to improving fibrosis by modulating adverse inflammatory events (Ong et al., 2015; Choo et al., 2017). When these profibrotic interplay and crosstalk between different resident and non-resident immune and non-immune cells in the stressed heart outweigh the reparatory mechanisms, the consequential events ultimately lead to cardiac fibrosis. Abbreviations: MERTK, proto-oncogene tyrosine-protein kinase MER; NGAL, neutrophil gelatinase-associated lipocalin; IFNγ, interferon gamma; TNF-α, tumor necrosis factor-alpha; IL1β, interleukin-1 beta; IL6, interleukin 6; IL15, interleukin 15; IL12, interleukin 12; IL8, interleukin 8; IL17, interleukin 17; IL9, interleukin 9; IL4, interleukin 4; IL13, interleukin 13; MMP, matrix metalloproteinase; TIMP, tissue inhibitors of metalloproteinases; SMAD, suppressor of mothers against decapentaplegic; IRF3, interferon regulatory factor 3; MPO, myeloperoxidase; NET, neutrophil extracellular traps; GM-CSF, granulocyte–macrophage colony-stimulating factor; DAMP, damage-associated molecular pattern; Snail, zinc finger protein SNAIL1; SLC, solute carrier; MCP-1, monocyte chemoattractant protein-1; VEGF, vascular endothelial growth factor; EGF, epidermal growth factor; EAR1, eosinophil-associated ribonuclease 1. Figure created using BioRender.

Specific localization of different immune cells in the heart suggests that they have specific interactions with resident cardiac cells. Cardiomyocytes, fibroblasts, and endothelial cells not only express receptors that recognize inflammatory mediators from inflammatory cells but also produce growth factors, cytokines, and chemokines to which leukocytes respond. Mast cell-derived tumor necrosis factor (TNF) activates endothelial cells (Frangogiannis et al., 1998a), IL-6 from cardiomyocytes activates neutrophils via intercellular adhesion molecule 1 (ICAM1) expression (Gwechenberger et al., 1999), IL-17 from T cells stimulates cardiac fibroblasts (Wu et al., 2014), and fibroblast-derived granulocyte–macrophage colony-stimulating factor (GM-CSF) induces the production and recruitment of myeloid cells (Anzai et al., 2017). On the other hand, macrophage-derived TGFβ, vascular endothelial growth factor (VEGF), and IL-10 promote collagen production, neo-angiogenesis, and resolution of inflammation (Nahrendorf et al., 2007; Hilgendorf et al., 2014) (Figure 2).

Newer subsets of immune cells and novel roles of different inflammatory cells are being revealed with ongoing research. Leukocytes and their products are gaining focus in the context of normal physiological as well as pathological fibrotic events. A brief discussion of the major contributors to cardiac fibrosis is provided in the following section.

### 2.1 Monocytes and macrophages in cardiac fibrosis

Cardiac macrophages are part of a steady-state cell network that contributes not only to forming a repertoire of immune cells but also to maintaining the mechanically strenuous and energy-intense pumping function of the heart. They electrically couple to cardiomyocytes through connexin 43 (CX43, also known as GJA1)-containing gap junctions in normal mouse and human hearts (Fernández-Ruiz, 2017). Macrophage ablation results in cardiac pathologies such as progressive atrioventricular block and conduction abnormalities in the atria and ventricles (Hulsmans et al., 2017)**.** Moreover, macrophages help in cell and matrix turnover and regulate angiogenesis and matrix deposition and removal in cardiac pathologies (Guo et al., 2018; Simões et al., 2020; Li et al., 2023a). Hence, spatiotemporal alteration and phenoconversion of macrophage populations in cardiac fibrosis not only remodel the structure but also cardiac rhythmicity (Figure 2).

#### 2.1.1 Subsets of cardiac macrophages

Macrophages and monocytes regulate fibrotic responses across many tissues (Wynn and Ramalingam, 2012)**.** The myocardium of adult mammals typically has a restricted number of resident macrophages (Gersch et al., 2002; Heidt et al., 2014; Mylonas et al., 2015) which play a role in maintaining cardiac homeostasis (Hulsmans et al., 2017)**.** Moreover, the development of sophisticated tools, such as single-cell RNA sequencing, has found evidence of heterogeneous and ontogenetically diverse macrophages in the heart. At least seven subsets of cardiac and pericardial macrophage populations have been identified in the infarcted heart with unique spatiotemporal dynamics and morphologic characteristics (Bajpai et al., 2018; Wei et al., 2023). Tissue-resident cardiac macrophages are principally divided based on the expression of CC-chemokine receptor 2 (CCR2). CCR2^-^ macrophages are of embryonic origin, and they seed the cardiac tissue during embryonic and early postnatal development. They support the development of coronary vasculature (Leid et al., 2016), cardiac regeneration, and electrical conduction within the atrioventricular (AV) node (Aurora et al., 2014; Lavine et al., 2014; Hulsmans et al., 2017)**.** On the other hand, adult hematopoietic lineages produce CCR2^+^ macrophages, which facilitate neutrophil extravasation and monocyte recruitment (Li et al., 2016) and initiation of inflammation within the diseased heart (Epelman et al., 2014b; Heidt et al., 2014; Epelman et al., 2015; Bajpai et al., 2019)**.** Another recent study reported the existence of a GATA6^+^ population of macrophages with an anti-fibrotic role within the pericardial cavity (Deniset et al., 2019). The dynamics of macrophage lineages change over time depending on the injurious stimuli and try to adopt changes to cope with the altered microenvironment. Thus, different subsets of macrophages contribute to the maintenance as well as pathological events of the heart.

#### 2.1.2 Macrophage plays an integral role in cardiac fibrosis

The macrophage heterogeneity influences the outcome of myocardial injuries in neonatal and adult hearts. The neonatal heart contains only CCR2^-^ cardiac resident macrophages, while the adult heart contains both CCR2^-^ and CCR2^+^ macrophage populations (Aurora et al., 2014; Lavine et al., 2014)**.** Following an injury, for example, after an MI, the neonatal heart expands CCR2^-^ macrophages, while the adult heart expands CCR2^+^ macrophages. In the adult heart, monocyte-derived CCR2^+^ macrophages are recruited by the inflammatory signal transduction pathways, DAMPs, and other chemokine-driven pathways and replace the CCR2^-^ population (Dewald et al., 2005; Frangogiannis et al., 2007). Upon recruitment, the CCR2^+^ population continues the phagocytic activities and promotes inflammation by activating the inflammatory bodies, by pattern recognition receptor (PRR) signaling, and by producing cytokines (Epelman et al., 2014a; Heidt et al., 2014; Sager et al., 2016; Bajpai et al., 2019; Chen et al., 2023; Isidoro and Deniset, 2023). These macrophages play a role in regulating inflammation, promoting fibrosis, facilitating matrix remodeling, supporting angiogenesis, and contributing to the process of regeneration. They release different cytokines, such as interleukin-1β, tumor necrosis factor-alpha (TNF-α), interleukin-6 (IL-6), interleukin-16 (IL-16), interleukin-18 (IL-18), transforming growth factor-β (TGF-β1), platelet-derived growth factor (PDGF), matrix metalloprotease-9 (MMP-9), and tissue inhibitor of metalloproteinase 1 (TIMP 1), and induce alpha-smooth muscle actin (αSMA), lysyl oxidase (LOX), and collagen type 1 alpha 2 (Col1a2) expression in fibroblasts, thus exhibiting profibrotic activity (Lindsey et al., 2006; MeléNdez et al., 2010a; Tamaki et al., 2013; de Couto et al., 2015; Frangogiannis, 2015; Chen and Frangogiannis, 2017; Honold and Nahrendorf, 2018; Liao et al., 2020; Moskalik et al., 2022). On the other hand, the macrophages with a reparatory phenotype (CCR2^-^) release IL-10 after the acute phase and attempt to repair cardiac function (Krishnamurthy et al., 2009; Jung et al., 2017; Moskalik et al., 2022). Metabolic reprogramming further polarizes macrophages into pro-inflammatory (M1) or anti-inflammatory (M2) type and, in turn, this phenoconversion orchestrates the background of scarring or healing of the injured myocardium (Kim et al., 2021; Kang et al., 2023) (Figure 2). Moreover, subsets of myeloid cells can engage in the fibrosis process by being converted to fibroblast-like cells or differentiating fibroblasts into myofibroblasts (Meng et al., 2016; Sinha et al., 2018; Haider et al., 2019; Simões and Riley, 2022; Wu et al., 2022)**.**

### 2.2 Granulocytes in cardiac fibrosis

#### 2.2.1 Direct role of neutrophils in cardiac remodeling

The role of neutrophils in the regulation of fibrosis is context-dependent. Reperfusion of the post-ischemic myocardium promotes neutrophil infiltration, which exacerbates the pro-inflammatory response and contributes to the ischemia–reperfusion (I/R) injury of the ischemic border zone. They produce and release reactive oxygen species (ROS) and myeloperoxidase (MPO), resulting in the generation of cytotoxic aldehydes, oxidative stress, activation of enzymes degrading the ECM and causing cardiomyocyte apoptosis, and maladaptive remodeling (Vinten-Johansen, 2004; Vasilyev et al., 2005; Fröhlich et al., 2013; Koeth et al., 2013; Puhl and Steffens, 2019; Okyere and Tilley, 2020). However, neutrophils exhibit a strong association with acute inflammatory reactions in the myocardium and play a role in tissue repair by influencing the behavior of macrophages (Horckmans et al., 2017)**.** In a mouse model, neutrophils have been shown to direct macrophages toward a reparative phenotype through the secretion of neutrophil gelatinase-associated lipocalin (NGAL) which mediates efficient clearance of debris. Moreover, antibody-mediated depletion of neutrophils increases cardiac fibrosis and worsens cardiac function (Horckmans et al., 2017) (Figure 2). Therefore, in regulation of fibrosis and for favorable cardiac remodeling, a balanced neutrophil response plays a crucial role.

Studies have found a high plasticity potential of neutrophils and their roles in modulating the outcome of inflammatory events. Ma et al. (2016) reported that the temporal polarization of neutrophils toward pro-inflammatory N1 exacerbates left ventricle (LV) remodeling, whereas the anti-inflammatory N2 phenotype attenuates adverse LV remodeling. DAMPs released from necrosed myocytes stimulate N1 polarization by stimulating TLR4, whereas TGF-β1 inhibition attenuates pro-inflammatory neutrophils (Ma et al., 2016)**.**

The presence of neutrophils in the wounded region is transient since they are rapidly eliminated. Replacement of neutrophils with Ly6C^low^ macrophages is aligned with the transition from the inflammatory phase to the reparatory phase and decreased production of inflammatory cytokines, growth factors, and chemokines (Nahrendorf et al., 2007)**.** Therefore, the involvement of neutrophils in chronic cardiac fibrosis is restricted to the initial phases of fibroblast activation. According to a recent study, a noteworthy mechanism depends on neutrophils and can potentially contribute to age-related cardiac fibrosis. Within the core of an aging organism, the stimulation of ROS in neutrophils can initiate the creation of neutrophil extracellular traps (NETs). This process is facilitated by activation of the peptidyl arginine deiminase 4 enzyme (PAD4). *In vivo*, tests indicate that the production of NETs through the involvement of PAD4 plays a role in the development of interstitial fibrotic alterations and the onset of left ventricular diastolic dysfunction (Martinod et al., 2017) (Figure 2).

#### 2.2.2 Contribution of basophils to cardiac fibrosis

Because of their rarity, basophils have long been overlooked in immunological research. Basophils circulate in the bloodstream under homeostatic conditions, but they infiltrate tissues during inflammation (Miyake et al., 2022)**.** Despite their low numbers, basophils affect the accumulation of myeloid cells and influence cardiac remodeling. Sicklinger et al. (2021) showed that basophil depletion promoted a shift from Ly6C^low^ macrophages with reparatory phenotype toward inflammatory Ly6C^hi^ monocytes in the infarcted myocardium. Induction of IL-4 and IL-13 by glycoprotein IPSE/α-1 in basophils improves cardiac functions and post-MI cardiac healing (Sicklinger et al., 2021)**.** The role of basophils in cardiac fibrosis is further corroborated by the finding that low blood basophil counts are associated with increased scar size and poor outcomes in patients with acute MI (Miyake et al., 2022)**.** Moreover, IL-4 released from the infiltrating basophils acts on resident fibroblasts, triggers myofibroblast expansion, and leads to the production of connective tissue elements from myofibroblasts (Schiechl et al., 2016)**.**

### 2.3 Lymphocytes in cardiac fibrosis

#### 2.3.1 B cells in cardiac fibrosis

The adaptive immune cells, B and T lymphocytes, are found in small numbers in a normal physiological heart but increase following injury (Zouggari et al., 2013; Wang et al., 2019). The B-cell population in the human heart is divided between the intravascular space and the interstitial space (Bermea et al., 2022) and further divided into subgroups. B1 and B2 cells contribute to the innate immune response through secretion of IFN-γ, IL-6, and IL-17; promote CD4^+^ T-cell polarization; and regulate the mobilization of monocytes through the production of CCL7, whereas B_regs_ or B10 cells support immunologic tolerance, resolve the acute inflammatory response, and maintain the homeostasis of certain types of natural killer cells through the secretion of IL-10, IL-35, and TGF-β (Zouggari et al., 2013; Chimen et al., 2015; Shen and Fillatreau, 2015) (Figure 2).

Cardiac B cells in neonatal mice promote cardiomyocyte proliferation, angiogenesis, and regeneration of the heart and inhibit inflammatory responses, while adult B cells promote inflammation and impair cardiac function following myocardial injury (Zouggari et al., 2013; Tan et al., 2023). Depletion of neonatal B cells reduces cardiac regeneration and promotes fibrotic scarring in the post-MI heart, whereas B-cell depletion in adult mice inhibits myocardial fibrosis and improves cardiac function (Tan et al., 2023)**.** Moreover, activated B cells contribute to sustained immune system activation and myocardial inflammation, promote the synthesis of myocardial collagen types I and III, and damage the left ventricular ejection fraction (Mo et al., 2021)**.** Studies have found that B cells promote fibrosis through releasing inflammatory cytokines like IL-1β, IL-6, and TNFα, whereas depletion of B cells results in attenuation of collagen deposition following MI, transverse aortic constriction, and nonischemic cardiomyopathy (Yu et al., 2013; Cordero-Reyes et al., 2016; Ma et al., 2018)**.**

#### 2.3.2 Heterogeneity of T-cell populations in cardiac fibrosis

T-cell receptor engagement, antigenic stimuli, tissue microenvironment, and metabolic reprogramming shape the repertoire of T cells into that of T helper cells (Th1, Th2, Th9, Th17, and Th22), cytotoxic T lymphocytes (CTLs), regulatory T (T_reg_) cells, and natural killer T (NKT) cells (Zhang and Zhang, 2020)**.** Induction of T cells by cardiac DAMPs processed by antigen-presenting cells results in cardiotropism, transformation of cardiac fibroblasts, and maladaptive cardiac remodeling (Bayer et al., 2023) (Figure 2). An increasing body of literature indicates that distinct subpopulations of T lymphocytes play a substantial role in the direct stimulation of fibroblasts and progression of cardiac fibrosis (Nevers et al., 2015; Li et al., 2017; Abdullah and Jin, 2018)**.** One study proposed the presence of a binding interaction between activated Th1 cells and cardiac fibroblasts in the left ventricular pressure overload model. The interaction has the potential to trigger the synthesis of TGF-β by fibroblasts, resulting in the differentiation of fibroblasts into myofibroblasts (Nevers et al., 2017)**.** Furthermore, an increased number of Th2 cells in cardiac tissues are afflicted with fibrosis (Duerrschmid et al., 2015)**.** The behavior under observation can be attributed to the increased regulation of profibrotic cytokines, such as IL-4 and IL-13, which effectively enhance collagen synthesis by fibroblasts. The infiltration of Th17 cells into the fibrous myocardium has been suggested to be involved in the pathogenesis of autoimmune myocarditis, contributing to the progression of a fibrous myocardium (Baldeviano et al., 2010)**.** Moreover, T cells can stimulate fibroblast activation via their fibrogenic activity. The profibrotic impact produced by T lymphocytes suggests their involvement in the survival of cardiac elastocytes, resulting in the replacement of dead cells by fibrous tissues (Kallikourdis et al., 2017)**.**

#### 2.3.3 Emerging insights regarding the role of T cells in cardiac fibrosis

Although the precise influence of different T-cell subpopulations on the development of fibrosis remains unclear, a growing body of evidence indicates that the use of regulatory T cells (T_regs_) in cellular therapy holds promise in reduction of the incidence of myocardial infarction and consequently the ensuing fibrotic response (Kvakan et al., 2009; Tang et al., 2012)**.** T_regs_ could reduce the fibrogenic activity of macrophages and CD8^+^ T-cell depletion after experimental MI in mouse models, which reduces inflammation and preserves ventricular function (Weirather et al., 2014; Santos-Zas et al., 2021). Furthermore, Saxena et al. (2014) have reported that T_regs_ could impact the phenotype of fibroblasts. Moreover, they have been observed to secrete signals that inhibit fibrosis (Tang et al., 2012); however, the precise characteristics of these signals have yet to be elucidated. Notably, T_regs_ can secrete fibrous mediators, such as TGF-β (Taylor et al., 2006; Saxena et al., 2014; Wang et al., 2023). Hence, the regulatory role of these cells in the fibrotic response is likely dependent on the specific circumstances and the balance between fibrotic and anti-fibrotic cellular processes.

#### 2.3.4 Emerging role of NK cells in cardiac fibrosis

Natural killer cells (NK cells) are type-I innate lymphoid cells known for their role in the recognition and elimination of virus-infected and malignant cells and in limiting their spread (Chiossone et al., 2018). NK cells also modulate immune responses by reciprocally interacting with macrophages, dendritic cells, T cells, and endothelial cells and contribute to the regulation of cardiac diseases (Vivier et al., 2008; Ong et al., 2017). Sustained NK cell deficit in patients with coronary artery disease was associated with persistent low-grade inflammation in the heart (Backteman et al., 2013). As an unabated chronic inflammation plays a role in eventual fibrotic remodeling of the cardiac tissue, NK cells might play a potential role in repairing and maintaining tissue homeostasis by modulating the inflammatory cascades (Tosello-Trampont et al., 2017). Activated NK cells aggregate in the heart, release granzyme B and IFN-γ, and exhibit increased expression of activation markers such as CD69, TRAIL (tumor necrosis factor-related apoptosis-inducing ligand treatment), and CD27. Hyperactivation of NK cells suppresses eosinophil activation and causes eosinophil apoptosis in an anti-inflammatory milieu, leading to decreased cardiac fibrosis (Ong et al., 2015). Moreover, NK cells lower the expression of eosinophil-related chemokines, eotaxin 1 (CCL11), eotaxin 2 (CCL24), CXCL9, and CXCL10 by resident cardiac fibroblasts (Ong et al., 2015). In post-myocardial injuries, NK cells lower cardiomyocyte apoptosis, collagen deposition, and consequent fibrosis and promote neovascularization (Ayach et al., 2006; Boukouaci et al., 2014). However, the role of NK cells in MI still lacks full characterization, and more research studies are required to explore the potential of NK cells in limiting the deposition of collagen and development of cardiac fibrosis.

### 2.4 Mast cells in cardiac fibrosis

#### 2.4.1 Mast cells do more than allergic reaction

The myocardium of adult animals harbors a limited population of mast cells. Notably, the abundance of mast cells in big mammals, such as dogs, surpasses that observed in mice (Frangogiannis et al., 1999). Cardiac mast cells are multifaceted resident immune sentinel cells playing a pivotal role in fibrotic remodeling in response to various myocardial injuries (Legere et al., 2020; Jin et al., 2022). Heart failure has been found to correlate with elevated quantities of mast cells (Shiota et al., 2003; Wei et al., 2003; Somasundaram et al., 2005; Luitel et al., 2017; Guimbal et al., 2021). Mast cells recognize DAMPs through TLR and ST2 (interleukin 1 receptor-like 1 receptor) and release preformed mediators and synthesize and secrete cytokines, chemokines, and lipid mediators (Janicki et al., 2015; Shao et al., 2015; Jin et al., 2022). The precise mechanisms responsible for increasing mast cells in the fibrotic cardiac area have yet to be fully elucidated. The growth factor known as stem cell factor (SCF) is crucial in the recruitment, development, and proliferation of fully developed mast cells. Additionally, it has been suggested that SCF may contribute to the localized increase in mast cells in the heart, thereby influencing cardiac pathology (Frangogiannis et al., 1998a). The origin of mast cells that infiltrate the diseased myocardium could be derived from adipose tissue (Ngkelo et al., 2016). Though the role of mast cells in the angiogenic responses in hypoxic tissues and cardiac fibrosis is reported, it lacks a complete picture. The pathophysiological role of mast cells depends on the tissue microenvironment and can be both pro- or anti-fibrotic in nature (Legere et al., 2019) (Figure 2).

#### 2.4.2 Mast cells contribute to experimental cardiac fibrosis

Experimental data show that mast cell growth significantly impacts cardiac fibrosis progression (Levick et al., 2011; Levick and Widiapradja, 2018; Widiapradja et al., 2019). In a mouse model of left ventricular pressure overload, mice without mast cells showed decreased perivascular fibrosis, which is associated with decreased progression to decompensated heart failure (Hara et al., 2002). After pressure overload, mast cells accumulate in the artery, contributing to ventricular fibrillation through platelet-derived growth factor-A (PDGF-A) expression (Liao et al., 2010). In hypertensive rat models, the stabilization of mast cells reduces fibrotic cardiac remodeling by preventing myocardial infiltration by macrophages (Levick et al., 2009). On the other hand, cardiac fibroblasts showed a profibrotic phenotype in response to mast cell mediators in mice with cardiac-specific overexpression of TNF (tumor necrosis factor) (Zhang et al., 2011). The significant profibrotic effects of mediators derived from mast cells are supported by the findings that left ventricular diastolic dysfunctions are present in many patients with systemic activation disorders of mast cells (Kolck et al., 2007).

#### 2.4.3 Mast cell derivatives activate fibroblasts and promote fibrosis

Mast cells can store a diverse array of preformed fibrogenic mediators within granules alongside their capacity to generate cytokines and growth factors (Grützkau et al., 1998; Patella et al., 1998). These synthetically generated bioactive chemicals exhibit notable effectiveness in stimulating fibroblasts upon exposure to external stimuli. Following an injury, mast cell degranulation can be initiated through a range of mechanisms, such as the activation of the complement system, the formation of ROS, the activation of adenosine receptors, or the stimulation of cytokines (Frangogiannis et al., 1998a; Murray et al., 2004; Meléndez et al., 2010b). The process of degranulation results in the release of substantial quantities of fibrous agents, which have the potential to initiate or intensify the fibrous response, such as TNF-α (Frangogiannis et al., 1998a), TGF-β (Shiota et al., 2003), IL-4 (Kanellakis et al., 2012), and PDGF (Nazari et al., 2016). Nevertheless, it is important to acknowledge that fibrinogen production is not limited solely to mast cells. Various additional cell types, such as macrophages, lymphocytes, vascular cells, and myocytes, collectively contribute to the pathogenesis of cardiac fibrosis, as discussed earlier. The precise involvement of mast cells in this pathway has yet to be determined, although histamines, tryptases, and chymases can substantially influence the fibrotic process due to their distinct localization within mast cells (de Almeida et al., 2002) (Figure 2).

Chymases can generate angiotensin II (Urata et al., 1990), potentially making it a key mast cell-derived mediator in cardiac fibrosis. It has been proposed that over 75% of cardiac-specific angiotensin II in failing hearts may come from the chymase pathway, independent of ACE (angiotensin-converting enzyme) (Urata et al., 1990). This pathway remains unaffected by ACE inhibitors, potentially offering a mechanism for cardiac fibrosis progression despite ACE inhibition. Chymase might also participate in the fibrotic response by activating MMPs (Fang et al., 1997; Stewart et al., 2003). Both rodent and large animal studies on cardiac fibrosis emphasize the significance of mast cell chymases, suggesting potential therapeutic avenues. For instance, chymase inhibition reduced fibrosis and diastolic dysfunction in a dog model of tachycardia-induced heart failure (Matsumoto et al., 2003), decreased cardiac fibrosis and MMP expression in a porcine reperfusion infarction model (Oyamada et al., 2011), and attenuated interstitial fibrosis while preventing diastolic dysfunction in a rat non-reperfused myocardial infarction model (Kanemitsu et al., 2006). Tryptase, the most abundant product of human mast cells, effectively activates fibroblasts by promoting proliferation (Ruoss et al., 1991) and collagen I synthesis (Cairns and Walls, 1997) through the protease-activated receptor (PAR)-2, leading to ERK-MAPK signaling activation (McLarty et al., 2011). Despite *in vitro* evidence supporting the fibrogenic effects of tryptase and its expansion in fibrotic hearts (Frangogiannis et al., 1998b; Somasundaram et al., 2005), there is a lack of studies investigating the *in vivo* role of tryptases in fibrotic cardiac remodeling.

Although the available data generally indicate that mediators derived from mast cells play a role in the accumulation of fibrous tissue, certain experimental studies have proposed that mast cells might possess features that counteract fibrosis (Joseph et al., 2005). The specific processes responsible for this protective phenomenon are still not fully understood. A potential association may exist between the indirect effects of mast cells on the viability of cardiomyocytes or the expression pattern of growth factors (Ngkelo et al., 2016). Like macrophages, mast cells can modify the expression profile of growth factors and proteins in reaction to signals from the microenvironment and metabolic demand (Xiong et al., 2022). The data indicate a possible transition from a state that promotes fibrosis to one that inhibits fibrosis.

### 2.5 Dendritic cells in cardiac fibrosis

Dendritic cells (DCs) are novel players in various fibrotic diseases where they possess a central role as antigen-presenting cells to regulate the immune system and inflammatory response. Studies have reported an immunoprotective role of the infiltrated DCs in experimental post-MI healing (Nagai et al., 2014; Carvalheiro et al., 2020; Sun et al., 2021). A decreased number of DCs, associated with increased infiltration of macrophages, impaired reparative fibrosis, and cardiac rupture following MI, suggests a protective role of DCs in the cardiac healing process (Nagai et al., 2014). The myocardium harbors both conventional dendritic cells (cDC) and plasmacytoid dendritic cells (pDCs), which maintain the infiltration of other leukocytes in the injury site area. Infiltration of activated DCs into the injured myocardium mediates the regulation of monocyte and macrophage homeostasis in the infarct area (Naito et al., 2008). Depletion of bone marrow-derived CD11c^+^ cells resulted in sustained release of IL-1β, IL-18, TNF-α; a high level of MMP-9 activity; and a decreased level of IL-10 in a mouse model, which resulted in enhanced fibrosis (Anzai et al., 2012) (Figure 2).

Cardiac cDCs recruited by chemokine receptor CCR2 cause upregulation of cardiomyocyte hypertrophy and inflammation by advanced glycation end products (Cao et al., 2015). Santos et al. (2020) showed that tolerogenic DCs attenuated lymphoproliferation and increased the FOXP3^+^ CD4^+^ T_reg_ cells in a mouse model. These events led to diminished IFN-γ, IL-12, and Col1a2 and increased IL-10, resulting in improved cardiac remodeling (Santos et al., 2020). These studies support the role of DCs in post-injury cardiac remodeling; however, more studies are needed to provide a clearer mechanistic insight into how DCs influence fibrosis.

## 3 Metabolic regulation of cardiac homeostasis

### 3.1 Metabolic flexibility of the homeostatic heart

The normal adult heart derives approximately 70%–90% of ATP from the oxidation of fatty acids (FAs) and the remaining from the oxidation of glucose, lactate, ketone bodies, and certain amino acids. Mitochondrial oxidative phosphorylation generates most of the ATP required, whereas glycolysis and GTP formation in the TCA cycle provide only around 5% (Lopaschuk et al., 2010; Doenst et al., 2013). The coordinated FA oxidation enables the heart to maintain its ability to switch between available substrates. Moreover, FA produces the greatest ATP yield per 2-carbon energy substrates with the highest O_2_ consumption (Fillmore et al., 2014). On the other hand, glucose works as a flexible substrate as it provides ATP by cytoplasmic glycolysis and mitochondrial oxidation of the pyruvate derived from glucose metabolism. More importantly, glucose supplies ATP most efficiently in the ischemic and stressed myocardium (Tian et al., 2023). Glucose metabolism generates pyruvate, lactate, and acetyl-CoA, which are further used in the pentose phosphate pathway or replenish TCA cycle intermediates in the mitochondria. Thus, glucose plays a dynamic role in the overall cardiac metabolic balance.

Ketone bodies and amino acids have a minor contribution to the overall cardiac oxidative metabolism in a normal heart, but prolonged fasting, ketogenic diet, and poorly controlled diabetes increase the ketone body utilization by the heart *in vivo*, while lactate or ketone supplementation in the perfusate reduces the glucose and FA oxidation in isolated perfused hearts *ex vivo* (Jeffrey et al., 1995; Stanley et al., 2003; Wentz et al., 2010; Papazafiropoulou et al., 2021). Ketone bodies are readily metabolized by the heart, and β-hydroxybutyrate is predominantly oxidized in the heart. Ketones produce ATP with a median efficiency compared to FAs and glucose and hence are a major fuel for the heart with increased circulating ketone levels (Ho et al., 2021). On the other hand, the carbon skeleton produced on branched-chain amino acid (BCAA) metabolism, including acetyl-CoA, α-ketoglutarate, acetoacetyl CoA, succinyl CoA, pyruvate, fumarate, and oxaloacetate, is used in the processes of the TCA cycle to offset the energy demand (Newgard, 2012). The TCA cycle enzymes produce the reducing equivalents NADH and FADH_2_ within the mitochondrial matrix (Tymoczko et al., 2002) and act as a pivotal mechanism to interconnect the glycolytic, beta-oxidation, and amino acid oxidation pathways (Brandt U and Heinrich Pc, 2014).

Mitochondria not only generate energy but also contribute to cellular signaling, maintain redox equilibrium, and act as a hub for the interconnected metabolic pathways of different substrates (Schaper et al., 1985). Fatty acyl-coenzyme A (CoA) and pyruvate from FA and glucose metabolism, respectively, feed mitochondria, whereas lactate, ketone bodies, and amino acids get oxidized directly in the mitochondria. All the energy-yielding substrates converge on acetyl-CoA production via specific catabolic pathways, which ultimately enter the TCA cycle and accomplish the energy transfer through oxidative phosphorylation (Kolwicz et al., 2013; Passarella and Schurr, 2018)**.** In short, the healthy adult heart is metabolically flexible, with FAs being the predominant substrate, followed by lactate, ketone bodies, glucose, and BCAAs (Ng et al., 2023)**.** To meet its ATP demand for continual cardiac contraction and pumping activities, the heart can readily shift between different energy substrates.

### 3.2 Metabolic reprogramming in the stressed heart

During the early events of fibrogenesis, mitochondrial and cellular homeostatic signaling and metabolic balance experience both qualitative and quantitative derangements. A shift in substrate preference away from FAs toward more anaerobic substrates leads the energy-compromised organ to suffer from a progressive burnout, which causes further functional deterioration (He et al., 2022). Perfused hypertrophic rat hearts showed a decrease in FA oxidation and increased glucose utilization consistently when subjected to regional myocardial infarction (Remondino et al., 2000), pressure overload (Allard et al., 1994; Christe and Rodgers, 1994), or volume overload (el Alaoui-Talibi et al., 1992; El Alaoui-Talibi et al., 1997) *ex vivo*. During stressful conditions, substrate versatility and complex regulatory mechanisms contribute to metabolic flexibility by transcriptional regulation and post-translational modification of crucial proteins in different metabolic pathways.

Limited oxygen supply during ischemia suppresses aerobic glucose and FA oxidation. The activation of the oxygen-sensing pathway and the HIF-1α leads to the transcriptional upregulation of glycolytic enzymes (Del Rey et al., 2017; Taylor and Scholz, 2022; Woods et al., 2022). Along with the shift of cardiac substrate metabolism from FA oxidation to glycolysis, GLUT4 gets translocated toward the sarcolemma, FA transporter FAT/CD36 moves away from the sarcolemma, and the glycogen content decreases (Heather et al., 2013). On the other hand, import of glucose into the cells by GLUT-4 depends on insulin, while glycogenolysis, triggered by an increase in AMP and inorganic phosphate and a decrease in ATP levels, generates glucose as an alternative source. Though glucose is not the major metabolic substrate for cardiac tissue, its utilization increases during ischemia, increased workload, and pressure overload hypertrophy (Stanley et al., 1992; Leong et al., 2002; Nascimben et al., 2004; He et al., 2012; Bertrand et al., 2020). This evidence supports metabolic reprogramming in cardiac cells during altered cardiac states as well as a shift in the metabolic axis, which is dependent not only on the energy demand but also on endocrine and neurohormonal homeostasis. Moreover, the heart relies more on glucose as its primary energy source in the context of disorders like diabetes and metabolic syndrome. This metabolic alteration has been found to have consequential effects on fibrosis-related cellular processes (Doenst et al., 2013; Hartupee and Mann, 2017).

Upregulation of ketone body utilization is another feature of the ischemic and hypertrophied failing heart to cope with the injurious event. 3-Hydroxybutyrate (3-OHB) enhances the bioenergetic thermodynamics of isolated mitochondria in the context of low FA levels. Moreover, a mouse model lacking 3-OHB oxidation is less adaptive to ischemic insult and pressure overload and culminated in worsened heart failure and remodeling (Aubert et al., 2016; Bedi et al., 2016; Horton et al., 2019; Manolis et al., 2023) However, more research is needed on cardiac ketone body metabolism in the context of pathological fibrosis and heart failure to elucidate the role of ketone bodies in the altered cardiac microenvironment.

Though amino acids have little contribution as oxidative fuel, myocardial uptake of several amino acids increases as a consequence of metabolic remodeling in pathological conditions. Amino acids are used in oxidative stress due to their potential non-oxidative metabolism and low contribution to cellular acidification. Glutamate and glutamine have been found to prolong cellular function when converted to α-ketoglutarate, while asparagine and aspartate remove amine groups and excess TCA cycle intermediates (Wischmeyer et al., 2003; Liu et al., 2007; Alkan and Bogner-Strauss, 2019). Moreover, glutamine and glutamate are used in ischemic and hypertrophied hearts to produce ATP directly through substrate-level phosphorylation and safeguard the cardiac tissue from being damaged by free radicals and low pH (Drake et al., 2012). Though the heart shows metabolic flexibility in terms of substrate preference governed by plasma substrate and hormone levels in normal physiological conditions, this phenomenon is significantly affected during pathology and failure (Jiang et al., 2021; Shi and Qiu, 2022). All these events are brought about as a result of the injury, metabolic adaptation to the altered microenvironment, and energy supply–demand mismatch and lead to changes in the ECM and architectural organization of the cardiac tissue and culminate in fibrosis.

### 3.3 How metabolism dyshomeostasis contributes to cardiac fibrosis?

#### 3.3.1 Direct effect of metabolism on the heart: cardiac fibroblasts and cardiomyocytes

Highly regulated and interconnected networks of metabolic pathways not only provide the energy currency for the functional integrity of the heart but also maintain the structural and spatiotemporal homeostasis of the cardiac tissue. Different metabolic pathways perform predominant roles and orchestrate the background of metabolic reprogramming while adapting to different stages of physiological development or pathological conditions. Cardiac fibrosis is the endpoint of multifarious cardiovascular pathologies, such as ischemic and nonischemic heart failure, pressure and volume overloads, genetic cardiomyopathies, diabetes, and aging (Gibb et al., 2020).

The myocardium contains a complex and intricate consortium of cardiomyocytes, endothelium, fibroblasts, pericytes, and immune cells. Upon injury, these cells acquire a fibrogenic phenotype by upregulating the expression of fibrosis-related genes and exhibit matrix synthetic and remodeling profiles. Moreover, DAMPs from dead cardiomyocytes activate inflammation, and collectively with the inflammatory cytokines, TGF-β, and other mediators, these events contribute to cardiac fibrosis (Zeisberg et al., 2007; Zhang et al., 2015; Alex et al., 2023). However, correlative expression studies of ECM proteins attribute the cardiac fibroblasts as the primary cell type responsible for myocardial fibrogenesis. Cardiac fibroblasts comprise around 10%–20% of the total cell population in the heart (Pinto et al., 2016). The nature and activities of fibroblasts, their response to the altered microenvironment, and metabolic reprogramming define the course of cardiac remodeling following any insult (Ali et al., 2014; Kanisicak et al., 2016; Kaur et al., 2016).

Though quiescent cardiac fibroblasts derive energy from mitochondrial oxidative phosphorylation, activation of fibroblasts and their differentiation into myofibroblasts display a stark increase in aerobic glycolysis and lactate production (Gibb et al., 2020). Moreover, the early stages of cardiac fibroblast activation align with altered mitochondrial morphology (Xin et al., 2019), produce mitochondrial reactive oxygen species (mtROS) (Jain et al., 2013), and show features of mitochondrial Ca^2+^ uptake (Lombardi et al., 2019). Cardiac fibroblasts display remarkable plasticity in response to injurious stimuli, change their own behaviors, and adapt quickly to the altered environment by transitioning between differentiation states (Fu et al., 2020). Metabolic patterns are remodeled between the initiation of cardiac fibroblast activation and their full differentiation into myofibroblasts. Quiescent cardiac fibroblasts require ATP to acquire contractile phenotypes, while fully activated cardiac fibroblasts use amino acid synthesis for collagen production. Moreover, lactate, succinate, and other amino acids serve as stimulators of myofibroblast differentiation (Tian and Ren, 2023)**.**

Altered glycolysis along with increased glycolytic enzymes, such as hexokinase, phosphofructokinase-1 (PFK1), pyruvate kinase, and lactate dehydrogenase (LDH), have been reported in activated cardiac fibroblasts and fibrotic diseases of different organs (Kottmann et al., 2012; Xie et al., 2015; Ding et al., 2017; Selvarajah et al., 2019). Following MI or angiotensin II administration, cardiac fibroblasts showed enhanced glycolysis and glutaminolysis, promoted the myofibroblast phenotype, and exacerbated myocardial injury (Lombardi et al., 2019). On the other hand, inhibition of glucose oxidation during enhanced glucose breakdown enables cardiac fibroblast activation and leads to the accumulation of lactate in myofibroblasts, which ultimately promotes histone lactylation following MI to express genes with a reparatory phenotype (Wang et al., 2022). Furthermore, increased levels of lactate enhance cardiac fibrosis and worsen cardiac dysfunction by promoting endothelial-to-mesenchymal transition via the transcription factor Snail1 lactylation after MI (Fan et al., 2023). Myofibroblasts undergo a shift away from FA oxidation toward glutamine utilization and α-ketoglutarate (α-KG) production. Enhanced glutaminolysis augments α-KG biosynthesis and cardiac fibroblast activation and contributes to *de novo* collagen synthesis from differentiated myofibroblasts (Gibb et al., 2022a).

#### 3.3.2 Direct effect of metabolism on nonmyocytes: immune cells

Although the cardiomyocyte is the heart’s parenchymal cell, the healthy heart also contains large amounts of nonmyocyte cells that help the organ contract (Pinto et al., 2016)**.** Through paracrine factor secretion, modifications to the ECM, gap junction coupling, and nitric oxide (NO) generation (Travers et al., 2016; He et al., 2017; DeBerge et al., 2019; Humeres and Frangogiannis, 2019; Mouton and Hall, 2020), nonmyocytes directly contribute to cardiac contractility. According to Pinto et al. (2016), a significant proportion of nonmyocytes in the heart, approximately 10%, consist of cardiac immune cells, with the majority being macrophages. These resident immune cells play important roles in the maintenance of normal cardiac homeostasis, and their metabolic state, immune phenotype, and overall disease progression are inextricably interwoven with the cardiac microenvironment. Immune cells, immune effectors, and their interactions with the parenchyma and stromal components are critical for tissue homeostasis and response to acute and chronic stressful conditions. The expression of multiple pro and anti-inflammatory cytokines and the presence of activated macrophages, immunomodulatory regulatory T-cells (T_regs_), and other cell types in the cardiac tissue may have adaptive roles in tissue health and maintenance (Zaidi et al., 2021)**.** In the following section, the role of metabolic reprogramming and phenoconversion of immune cells in the context of cardiac fibrosis is discussed.

#### 3.3.3 Metabolism of cardiac immune cells during cardiac fibrogenesis

**FIGURE 3:**
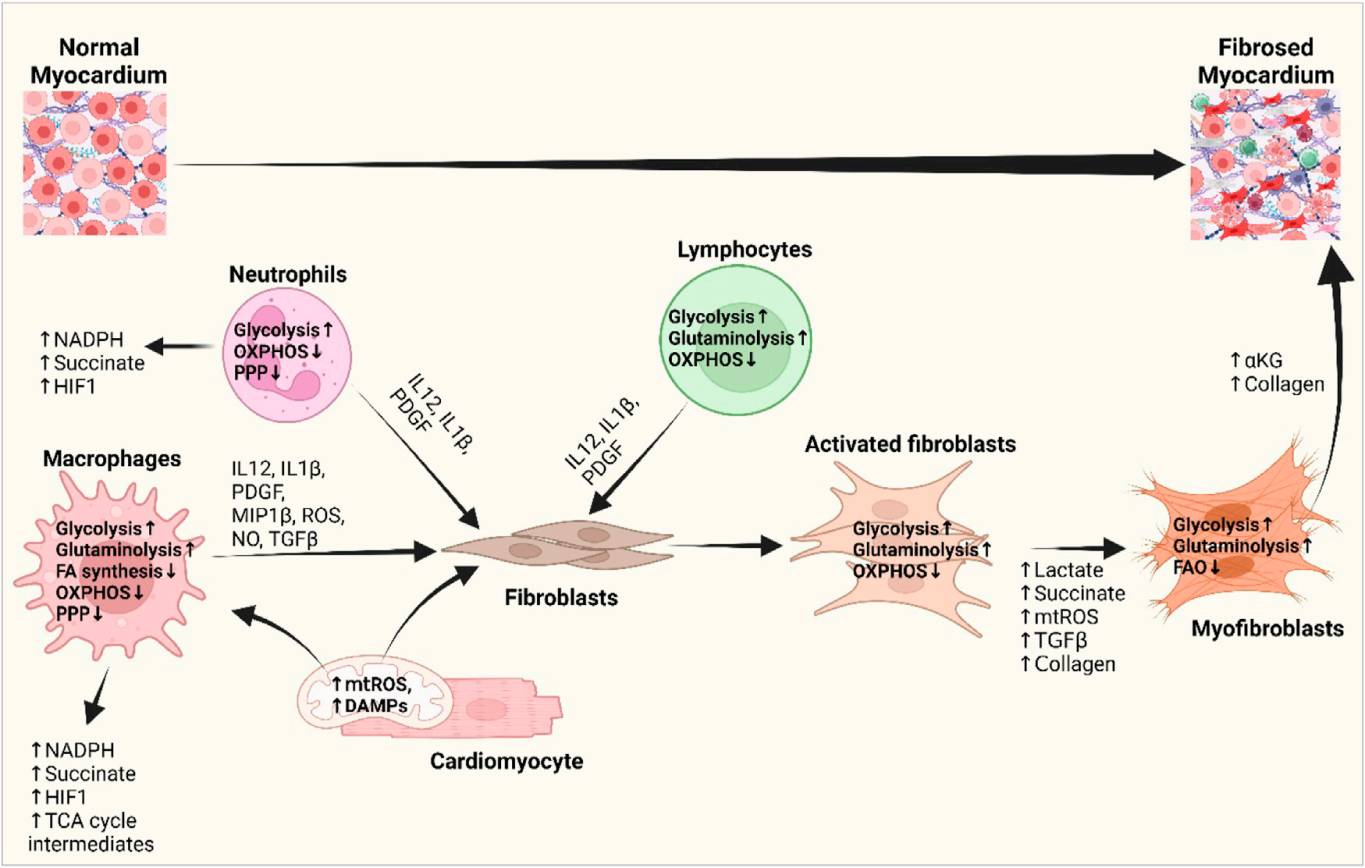
Interplay of inflammatory cells with fibroblasts and cardiomyocytes and their metabolic reprogramming in cardiac fibrogenesis. DAMPs from the damaged cardiomyocytes and mitochondrial ROS activate macrophages and fibroblasts and bring about metabolic changes to meet the altered metabolic demand. Increased glycolysis and amino acid utilization and decreased oxidative phosphorylation in macrophages, neutrophils, and lymphocytes promote the release of different growth and inflammatory factors, i.e., IL-1β, IL-12, PDGF, and ROS, which subsequently activate fibroblasts (Wang et al., 2008; Yoshikawa et al., 2022). Metabolic shifts promote the production of lactate, succinate, HIF1-α, and TCA cycle intermediates and stimulate myofibroblast differentiation. Myofibroblasts shift from fatty acid oxidation to glutaminolysis and promote αKG and collagen biosynthesis and fibrotic deposition in the heart (Gibb et al., 2022a; Fan et al., 2023). Abbreviations: DAMP, damage-associated molecular pattern; FA synthesis, fatty acid synthesis; HIF1, hypoxia-inducible factor 1; IL-1β, interleukin-1 beta; IL-12, interleukin-4; MIP1β, macrophage inflammatory protein-1 beta; mtROS, mitochondrial reactive oxygen species; NADPH, nicotinamide adenine dinucleotide phosphate; NO, nitric oxide; OXPHOS, oxidative phosphorylation; PDGF, platelet-derived growth factor; PPP, pentose phosphate pathway; TCA cycle, tricarboxylic acid cycle; TGF-β, transforming growth factor-beta. Figure created using BioRender.

Cardiac metabolism gets reprogrammed in pathology, evidenced by an augmented dependence on glucose metabolism and a reduced level of FA oxidation (Young et al., 2001; Akki et al., 2008; Kolwicz et al., 2013), and so do the immune cells in the heart. While myocytes exhibit metabolic adaptations in response to changes in ATP demand and substrate availability, immune cells primarily undergo metabolic reprogramming to facilitate phenotypic transition between distinct subsets (Zhang et al., 2019a). M1 macrophages and neutrophils depend on glycolytic metabolism to drive pro-inflammatory processes, including increased pentose phosphate pathway (PPP) activity to produce NADPH to synthesize pro-inflammatory lipid mediators (Zhang et al., 2019a) (Figure 3). The diminished activity and truncation of the TCA cycle with the succinate dehydrogenase enzyme leads to increased succinate dehydrogenase levels. This increase in succinate dehydrogenase levels subsequently triggers the activation of HIF-1α, which promotes the expression of genes associated with inflammation and glycolysis in macrophages. Upregulation of HIF-1α and glycolysis in macrophages following MI causes them to initially acquire a pro-inflammatory phenotype, generally referred to as the M1 phenotype, which plays a crucial role in myocardial remodeling (Mouton et al., 2018)**.** In contrast, polarized anti-inflammatory M2-type macrophages, expressing high levels of genes with a reparatory phenotype (Yan et al., 2013)**,** rely on oxidative phosphorylation, a complete TCA cycle, and the inhibition of glycolysis and the PPP (Zhang et al., 2019a). T lymphocytes inside the injured tissue undergo a metabolic reprogramming characterized by an increase in glycolytic metabolism and the presence of pro-inflammatory T-helper 17 (Th-17) cells (Wu et al., 2019)**.** The scope of promoting M2 macrophage and anti-inflammatory regulatory T-cell phenotype by metabolic intervention might nurture the promise of metabolic modulation in the progression of cardiac fibrosis (Zhang et al., 2019b; Zhao et al., 2020; Pérez and Rius-Pérez, 2022; Taylor and Scholz, 2022)**.**

##### 3.3.3.1 Reprogramming in glucose metabolism

The mounting of an immune response and functional reprogramming within a cell is associated with innate metabolic changes (O'Neill and Hardie, 2013). One of the most well-recognized changes is the activation of anerobic glycolysis, which is a common feature of inflammatory activation of activated macrophages (Rodríguez-Prados et al., 2010); dendritic cells (DCs) (Krawczyk et al., 2010); natural killer (NK) cells (Donnelly et al., 2014); B cells (Doughty et al., 2006); effector subsets of T cells: Th1, Th2, and Th17 (Michalek et al., 2011; Shi et al., 2011); and memory T cells (Gubser et al., 2013)**.** Upregulation of glycolysis forces them toward the pro-inflammatory phenotype (Andrew et al., 2014; Freemerman et al., 2014), while inhibition of glycolysis inhibits immune cell activation and drives them to polarize toward macrophages with a reparatory phenotype and T cells (Soto‐Heredero et al., 2020)**.**

The glycolytic phenotype shows increased expression of glycolytic enzymes and offers the immune cell a survival advantage in hypoxic environments as well as provides the Kreb’s cycle intermediates to produce inflammatory cytokines (Abhishek et al., 2015)**.** For example, the glycolytic enzyme hexokinase 1 has been shown to directly interact with and activate the NLRP3 inflammasome (Finucane et al., 2019; Baik et al., 2023)**.** Activation of NLRP3 is strongly linked with fibrosis, which drives the differentiation of fibroblasts into myofibroblasts by chronic upregulation of IL-1β and IL-18 and subsequent autocrine signaling that maintains an activated inflammasome (Artlett, 2022). Moreover, PDGF-BB, IL-12, IL-1β, and MIP-1β (macrophage inflammatory protein-1 beta) from immune cells promote the glycolytic process in fibroblasts which provide more ATP and biosynthetic intermediates for excessive production of the ECM, while inhibition of glycolysis attenuates fibroblast activation and cardiac fibrosis (Chen et al., 2021a; Feng et al., 2023)**.** Furthermore, enhanced glycolysis in immune cells can lead to increased production of ROS, contributing to oxidative stress and tissue injury, both of which are implicated in cardiac fibrosis (Harvey et al., 2014; Dhalla et al., 2022)**.** Hence, increased glycolysis is considered a hallmark of metabolic reprogramming in most immune cells undergoing rapid activation in response to stimulation of pattern recognition receptors, cytokine receptors, or antigen receptors which amplify the production of inflammatory cytokines (Figure 3). Thus, the complex interplay between the immune system and fibrotic processes in the heart could be used to modulate the fibrogenic cascades in the heart.

##### 3.3.3.2 Reprogramming in the TCA cycle

The TCA cycle and oxidative phosphorylation are intact in M2 macrophages and most T-cell subsets, whereas in effector T cells, there is a shift away from the TCA cycle, and in M1 macrophages, the TCA cycle breaks down at two sites: after citrate and after succinate (Abhishek et al., 2015; Liu et al., 2021)**.** As the TCA cycle disrupts in activated macrophages, the mitochondrial accumulation of citrate and succinate feeds into oxidative pathways to generate key effector molecules and metabolites in macrophages. Production of nitric oxide (NO), prostaglandins, IL-1β, and stabilization of HIF1α promote immune function (Infantino et al., 2011; Tannahill et al., 2013)**.** The chronicity of ongoing residual inflammation in cardiac tissue and activated immune cells play crucial roles in the fibrotic deposition of the ECM in the heart. The intermediates of the TCA cycle contribute to the modulation of the metabolic phenotype of immune cells, which consequently promotes fibroblast activity and ultimately exerts a fibrogenic effect on the heart. As the TCA cycle occurs in the mitochondrial matrix, the integrity of mitochondria as well as mitochondrial substrates during stressful conditions play roles in immune regulation as well (Martínez-Reyes and Chandel, 2020).

TCA cycle metabolites such as citrate, succinate, fumarate, oxaloacetate, α-KG, and L-malate accumulate in the cells with mitochondrial stress and link cellular metabolism to innate leukocyte responses and fibrosis (Patil et al., 2019; Ryan et al., 2019; Wu et al., 2023)**.** Transport of mitochondrial citrate by the citrate carrier SLC25A1 (solute carrier family 25 member 1) to the cytosol is upregulated in M1 macrophages in an NF-κB- or signal transducer and transcription (STAT)-dependent manner, which promotes NO, ROS, and prostaglandin E2 (PGE2) production, while inhibition of the citrate carrier reduces the inflammation (Infantino et al., 2011; Infantino et al., 2014; Williams and O'Neill, 2018). Increased glycolysis leads to increased production of α-KG from the TCA cycle to support collagen synthesis and might promote fibrosis (Henderson and O'Reilly, 2021) (Figure 3).

A key link between the TCA cycle (mitochondria) and fibrosis is oxidative injury, where increased ROS and mitochondrial DAMPs induce TGF-β expression in macrophages and fibroblasts and consequently induce myofibroblast differentiation, NLRP3 inflammasome activation, alter MMP/TIMP balances, and set off signaling cascades triggering fibrosis (Liu and Gaston Pravia, 2010; Ung et al., 2021)**.** Furthermore, mitochondrial dysfunction combined with energy deficiency-driven activation of CPI (carnitine palmitoyltransferase I) results in a substantially greater content of long-chain (LC) acylcarnitines. At high concentrations, LC acylcarnitines impede oxidative phosphorylation, causing mitochondrial membrane hyperpolarization and increasing the formation of ROS in cardiac mitochondria (Tominaga et al., 2008; Liepinsh et al., 2016)**.** Metabolic reprogramming of the TCA cycle in immune cells might provide mechanistic insights to modulate the fibrogenic events in cardiac remodeling.

##### 3.3.3.3 Reprogramming in lipid metabolism

FA oxidation and FA synthesis have opposing roles in the immune system. Inflammatory signals drive FA synthesis, immune cell proliferation, and inflammatory cytokine production, whereas non-inflammatory and tolerogenic immune cells prefer FA oxidation and the production of suppressive cytokines, leading to inhibition of inflammation. Effector T cells and activated macrophages show enhanced lipid synthesis, whereas M2 macrophages, regulatory T cells, and memory T cells show FA oxidation, which limits their growth and allows them to persist (Posokhova et al., 2008; Ecker et al., 2010; Michalek et al., 2011; Feingold et al., 2012) (Figure 3).

Accumulation of intracellular FAs stimulates IL-1α production in foam cells, leading to increased inflammation. M1 polarization is induced by IFNγ and LPS and acquires an inflammatory phenotype via glycolytic metabolism, whereas M2 polarization is promoted by IL-4, which induces FA oxidation by STAT6 and PPARγ-co-activator 1β (PGC1β). Overexpression of PGC1β attenuates M1 polarization even in the presence of IFNγ and LPS (Vats et al., 2006; Huang et al., 2014; Nomura et al., 2016). Moreover, intracellular accumulation of lipids and lipid intermediates leads to lipotoxicity, culminating in alterations in the structural morphology and impaired cardiac function. Lipotoxicity has the potential to induce apoptosis in cardiomyocytes by the augmentation of ROS production and endoplasmic reticulum stress, ultimately leading to the development of heart failure (Zlobine et al., 2016)**.**

Activation of NF-κB through TLR4 signaling induces SREBP (sterol receptor element-binding protein) expression and promotes lipid synthesis, which induces the cleavage and maturation of pro-IL-1 and pro-IL-18 and promotes the M1 macrophage phenotype (Im et al., 2011)**.** On the other hand, liver X receptor (LXR) upregulation and AMPK-enhanced β-oxidation of lipids induce the anti-inflammatory phenotype of immune cells (Zelcer and Tontonoz, 2006)**.** Moreover, altered lipid availability contributes to mitochondrial dysfunction and changes the macrophage phenotype. Proliferation of T cells depends on glycolysis and β-oxidation, whereas their activation depends on *de novo* lipogenesis. Lipogenesis also contributes to differentiation of Th17 cells to T_regs_. Activated T cells shift their dependence from lipid metabolism to glucose breakdown (Shyer et al., 2020)**.** As phenoconversion of immune cells essentially influences fibroblasts and myofibroblasts in the heart, modulation of FA metabolism by means of IL-10, AMPK, mTOR, or TLR signaling holds promise in intervening in cardiac fibrosis (Sag et al., 2008).

##### 3.3.3.4 Reprogramming in amino acid metabolism

The availability and metabolism of various amino acids play important roles in immune function, of which glutamine, arginine, and tryptophan are the most important. Adequate glutamine is used for the induction of IL-1 and NO production through feeding into arginine synthesis. Inadequate glutamine supply impedes cytotoxic macrophages from producing NO *in vitro*. Glutaminolysis promotes glycolysis via the α-KG/mTOR/HIF-1α pathway as well as contributes to amino acid synthesis and lipid metabolism and promotes ECM production in fibroblast and myofibroblast persistence (Gibb et al., 2022b)**.**

α**-**KG produced via glutaminolysis feeds OXPHOS and FA oxidation and promotes M2 polarization of the macrophages through Jmjd3 (Jumonji domain-containing 3)-dependent demethylation of H3K27 and attenuates the M1 phenotype by inhibiting IKK activation through PKH (prolyl hydroxylase domain) (Bai et al., 2019; Jia et al., 2019)**.** Moreover, glutamine supports the M2 phenotype by UDP-GlcNAc (glutamine–UDP-N-acetylglucosamine) synthesis via the hexosamine biosynthetic pathway. On the other hand, succinate synthesized via glutamine-dependent anaplerosis or the γ-aminobutyric acid (GABA) shunt promotes polarization of M1 macrophages (Ren et al., 2019). This evidence suggests that the cytotoxic function of macrophages could be modified by modulating the amino acid metabolism. Both T-cell and B-cell activation as well as the balance between effector T cells and T_reg_ cells depend markedly on glutamine usage. Loss of transporter protein ASCT2 (alanine–serine–cysteine transporter type-2) was found to reduce glutamine level in the cells and cause a defect in effector T-cell function by reducing mTORC1 activity in T cells (Crawford and Cohen, 1985; Carr et al., 2010; Le et al., 2012; Nakaya et al., 2014).

Arginine plays a dual role in immune activation. The flux of arginine into the NO synthesis pathway produces NO by inducible nitric oxide synthase (iNOS) and promotes inflammatory M1 macrophages, whereas the arginase pathway promotes tolerant immune responses and often is associated with wound healing. mTOR signaling regulates numerous events that are crucial for T-cell and monocyte differentiation (Weichhart et al., 2015)**.** mTORC1 activity in T cells is suppressed in arginine-depleted *in vitro* cultures (Cobbold et al., 2009). Arginase 1 expression in macrophages limits the inflammatory potential of effector Th2 cells and suppresses fibrosis. Moreover, macrophage-specific expression of arginase-1 promotes inflammation and fibrosis by enhancing L-proline, polyamine, and Th2 cytokine production (Pesce et al., 2009). The metabolic fates of the products of arginase and arginine deaminase in the immune cells suggest that arginine metabolism plays a key role in inflammation.

Extracellular amino acids support the energy-intensive T-cell activation process and contribute to immune regulation. Reduced extracellular amino acids, i.e., leucine, during ischemia, impair T-cell mobilization and mTOR-dependent Th1 and Th17 differentiation (Sinclair et al., 2013)**.** Moreover, an excessive amount of BCAA impairs mitochondrial function. This impairment is characterized by the disruption of the mitochondrial membrane potential and the opening of the mitochondrial permeability transition pore. The accumulation of branched-chain keto acids (BCKAs) from the degraded BCAA facilitates ROS generation (Zhao et al., 2023)**.** The metabolites, ROS, and oxidative stress crosstalk with fibroblasts activate profibrotic cascades, alter the turnover of the ECM, and ultimately shift the balance toward fibrosis. Manipulation of the cooperativity among cells for production of substrates for collagen synthesis gives us an insight into treating cardiac fibrosis (Cowling et al., 2019; Molek et al., 2021; Raziyeva et al., 2022)**.**

##### 3.3.3.5 Reprogramming in the pentose phosphate pathway (PPP)

Glycolysis feeds the PPP, which allows the diversion of intermediates from the glycolytic pathway toward the production of nucleotide and amino acid precursors as well as generates reducing equivalents of NADPH, which has an important role in the maintenance of a favorable cellular redox environment. Macrophages and neutrophils use NADPH for rapid ROS production to clear the insulting agent as well as for the induction of antioxidants to prevent excessive tissue damage (O'Neill et al., 2016)**.** The role of the PPP in immune cell activation, ROS production, and cell polarization has been found crucial in the study of sedoheptulose kinase carbohydrate kinase-like protein (CARKL) on macrophages. CARKL limits the flux through the PPP, and its suppression directs macrophages toward the M1 phenotype (Haschemi et al., 2012; Sun et al., 2020b)**.** NADPH generated by the PPP is used in the regeneration of antioxidants such as glutathione, which plays a crucial role in protecting cells from oxidative damage associated with inflammation and fibrosis.

## 4 The interplay between the immune system and metabolism in cardiac fibrosis

Is cardiac fibrosis an endpoint of the derangement of the normal immune physiology and metabolism of the heart? Various factors can contribute to the development of fibrosis, with one notable factor being the interplay between immune cells and metabolic pathways. However, the relationship between metabolic changes and immune responses during fibroblast-to-myofibroblast transition remains unclear. The crosstalk between immune cells and metabolic pathways is a complex and dynamic interaction that plays a crucial role in various physiological and pathological processes. Immune cell activation and function are intimately linked to metabolic pathways (Chi, 2022)**,** allowing cells to meet the energy demands required for their activities. The metabolic processes initiated by immune cells have the potential to impact the activation of fibroblasts and the subsequent buildup of the ECM through their influence on cytokine production and tissue healing mechanisms, hence facilitating development of fibrosis (Czubryt, 2019; Mouton and Hall, 2020; Eming et al., 2021; DeBerge et al., 2023; Hara and Tallquist, 2023)**.** Macrophages undergo phenotypic shifts from an M1 state to an M2 state. These transitions are accompanied by distinct metabolic profiles, including glycolysis and oxidative phosphorylation**,** which contribute to the respective activities of the macrophages (Wynn and Ramalingam, 2012) (Figure 3). Similarly, activated T cells undergo a metabolic shift from oxidative phosphorylation to glycolysis, facilitating the prompt energy generation and biosynthesis required for proliferation and effective functioning (Figure 3).

The impact of metabolic pathways, including the TCA cycle and FA oxidation, on the development and function of immune cells and fibroblasts provides newer perspectives on different metabolites in fibrotic diseases (MacIver et al., 2013)**.** An illustration of this phenomenon involves the utilization of metabolites, such as succinate, fumarate, and itaconate, as important signaling molecules that modulate physiology and pathology and regulate intercellular communication within the immune system (MacIver et al., 2013; Tannahill et al., 2013; Mills et al., 2016; Mills et al., 2018; Hooftman et al., 2023)**.** These metabolites can influence various immune cell populations, including regulatory T cells. The influence of metabolic pathways on the differentiation and function of T_reg_ has been reported. Maintenance of T_reg_ suppressive function relies on FA oxidation, underscoring the significance of metabolic signals in regulating the immune system (MacIver et al., 2013)**.** The phenomenon of metabolic reprogramming enables immune cells to adjust their energy and biosynthetic requirements in response to varying activation conditions and leads the cells of the surrounding microenvironment to initiate fibrosis. The role of mTOR in integrating nutritional availability and metabolic conditions to govern the differentiation and responsiveness of T cells suggests a strong interconnection between the activation and functionality of immune cells and several metabolic pathways (van der Windt et al., 2012) (Figure 3).

**FIGURE 4:**
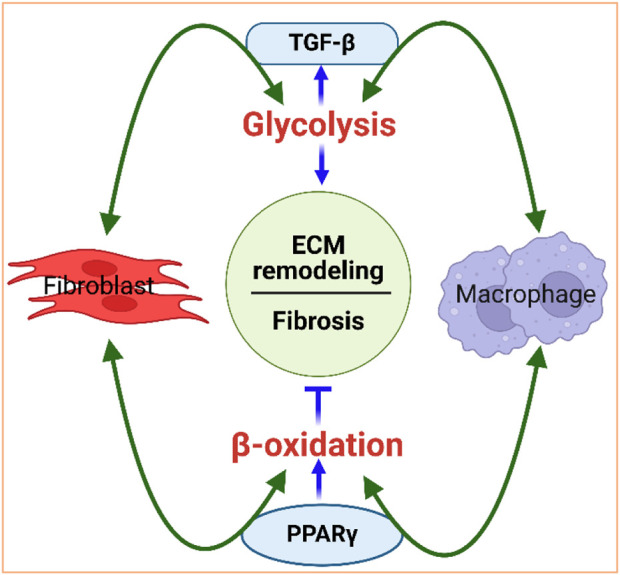
Interdependence of metabolism and ECM remodeling. Fibroblasts and macrophages work in concert to regulate the ECM. They are the primary cell types that mediate collagen internalization and degradation. For instance, fibroblasts and macrophages activate glycolysis, via TGF-β signaling, and promote fibrosis. Glycolysis, in turn, can increase TGF-β, further activating fibroblasts and macrophages. Fibroblasts and macrophages also activate beta-oxidation via PPAR signaling to promote degradation of the ECM. PPARγ can control macrophage polarization to either pro-inflammatory M1 or to anti-inflammatory M2 macrophages. Figure created using BioRender.

The interaction between metabolism and the fibrotic response is bidirectional, with metabolism playing a causal role in dictating cellular signaling and the effector functions of fibroblasts and immune cells. For example, TGF-β1, a profibrotic molecule expressed by activated fibroblasts that contributes to ECM remodeling (Kawaguchi et al., 2011; Mouton and Hall, 2020; Farah et al., 2021; Francisco et al., 2021), is a strong activator of glycolysis (Jiang et al., 2015) and immune response (Li et al., 2006). Conversely, glycolysis is involved in the activation of fibroblasts and is a critical regulator of TGF-β1 and collagen synthesis in cardiac fibroblasts (Singh et al., 2008)), suggesting that glycolysis upregulation is important in ECM remodeling, with TGF-β1 playing a potent role as a metabolic regulator of cardiac fibrosis (Figure 4). Similarly, lipid metabolism is also a key player in regulating ECM homeostasis and fibrosis. Peroxisome proliferator-activated receptor (PPAR), a major facilitator of FA oxidation, promotes fibroblast-mediated as well as macrophage-mediated degradation of the ECM, reflecting the potential interconnection between lipid metabolism and ECM remodeling (Figure 4).

## 5 Targeting metabolism and immune response in cardiac fibrosis

**FIGURE 5:**
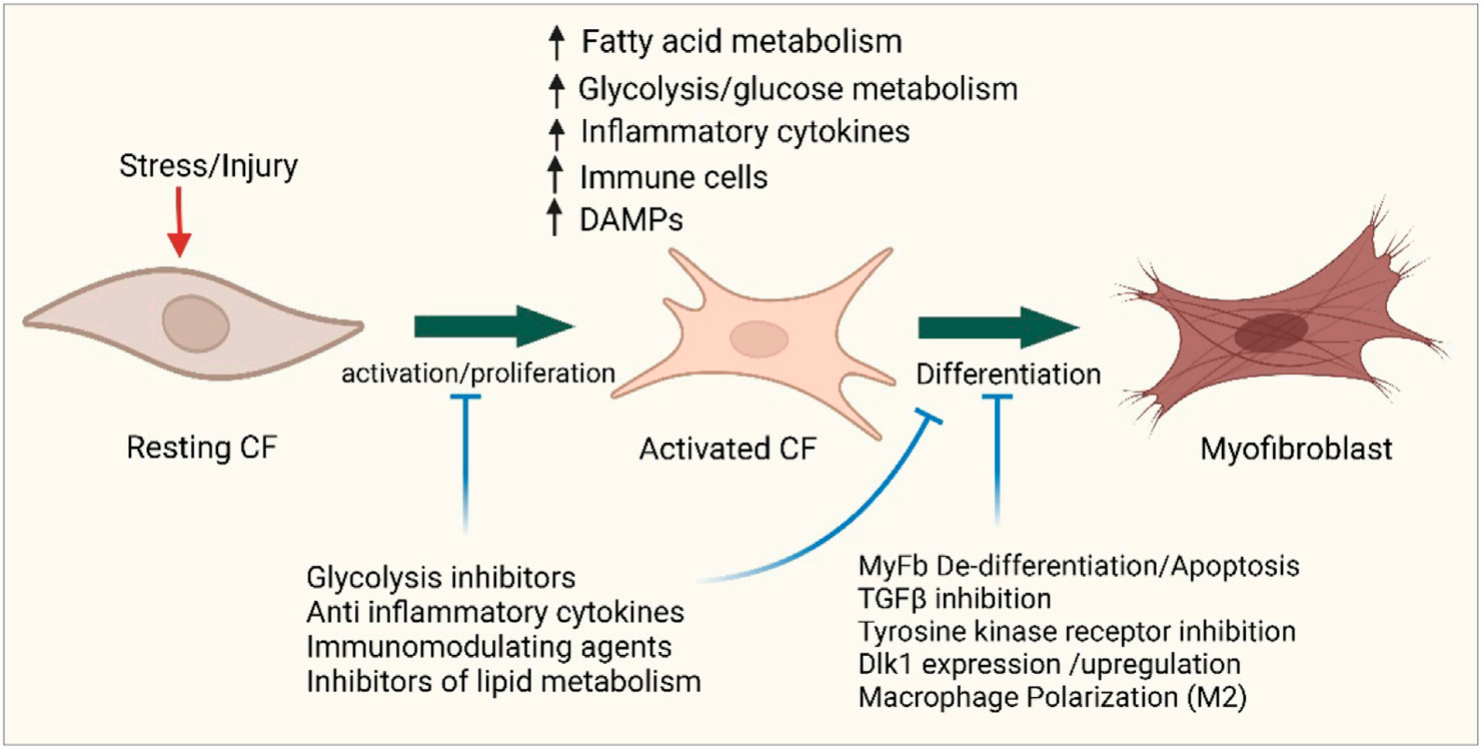
Targeting metabolism and immunity in cardiac fibrosis. Cellular map showing the sites of possible potential interventions in immune and metabolic pathways to improve the outcome of cardiac fibrosis. Figure created using BioRender.

Basic research has detailed the cellular and molecular mechanisms and signaling pathways driving this lesion; however, there is a clear lack of personalized anti-fibrotic strategies permissible for its effective treatment. Recent key findings implicating the innate and adaptive immune response and metabolic changes during the pathological transition of cardiac fibroblasts have tremendous potential and may offer opportunities to facilitate novel therapeutic strategies for the regulation of the treatment of fibrotic remodeling (Figure 5). For instance, given the remarkable success of immunotherapy in cancer treatment, the use of chimeric antigen receptor T cells (CAR T-cells) or modified T-cell receptors would be an ambitious approach. CAR T cells have been successfully used in the treatment of certain leukemias and solid tumors (Petrausch et al., 2012; Jogalekar et al., 2022)**.** Similarly, studies have shown that modified CAR T cells designed against activated fibroblasts improve myocardial fibrosis and cardiac function in mouse models of heart failure (Aghajanian et al., 2019; Rurik et al., 2022)**.**

Exploitation of metabolic pathways as possible targets for anti-fibrotic therapy should be considered, although it might be challenging to really determine the specific role of immune cell metabolism and its effect in cardiac fibrosis due to several factors acting in pathological condition; however, the influence of metabolism on immune cells remains quite unclear due to the spatiotemporal distribution of various immunological cells at various phases of cardiac fibrosis. Certain cell types which prove effective at a certain time might not have similar effects after some time. However, the combination of metabolic approaches could improve the prognosis of heart failure when combined with other treatment regimens such as ACE inhibitors, beta-blockers, and mineralocorticoid blockers (Stanley et al., 2005; McMurray et al., 2012; Yurista et al., 2022)**.** As such, the use of metabolic agents in cardiac fibrosis and heart failure could be beneficial. Some common metabolic agents that could be used target various metabolic processes, including FA/lipid metabolism and glucose/pyruvate metabolism (Rosano et al., 2015). For instance, niacin, also known as nicotinic acid, is involved in the lipid metabolism pathway and could alter the energy metabolism of cardiac fibroblasts by inhibiting β-oxidation and increasing glucose oxidation (Rosano et al., 2015). This increased glycolysis induction serves as a compensatory response to reduce mitochondrial oxidative metabolism and ATP production in heart failure (Lopaschuk et al., 2021; Ng et al., 2023) (Figure 5).

Glucose metabolism tightly regulates extracellular matrix production, and targeting glycolysis has shown exciting results in tackling fibrosis and other diseases. Glycolysis inhibitors have been reported as emerging therapeutic candidates in some cancer treatments (Akins et al., 2018; Chelakkot et al., 2023), while glycolytic reprogramming has been implicated as a regulator of pulmonary fibrosis (Xie et al., 2015; Li et al., 2022). This leads to the question of whether these inhibitors could be beneficial as well in the reversal of myofibroblasts in cardiac fibrosis (Figure 5). Although fibrosis is important for the normal healing process, excessive and continuous deposition of myofibroblasts in the injured myocardium is rather deleterious. It is therefore imperative that both immune responses and metabolic control should be adequately used in the arrest of fibroblast deposition at the adequate point in time during fibrosis. Chen et al. (2021a) reported that 2 deoxy-D-glucose, a glycolysis inhibitor, could alleviate cardiac fibrosis by reducing collagen volume and α smooth muscle actin expression. They also reported the reversal of fibroblast activation *in vivo* with glycolytic inhibitors. However, specific mechanisms through which these effects were achieved have not been well-documented. Another inhibitor of glycolysis, Kallistatin/Serpina3c, which inhibits the transcription of enolase, a key enzyme in glycolysis, has also been reported to inhibit cardiac fibrosis after MI, hence preventing fibroblast proliferation (Ji et al., 2022)**.** Several attempts have been made to use immunomodulators in the therapy of cardiac fibrosis, yet without much success, such as the use of TNF-α (Baumgarten et al., 2000; Rurik et al., 2021) or TGF-β neutralizing antibodies (Kuwahara et al., 2002) (Figure 5). This has been suggested to be associated with reduced efficacy of TGF-β inhibitors when used as monotherapy (Parichatikanond et al., 2020)**.** However, some other TGF-β inhibitors such as pirfenidone have proven effective in experimental conditions in the treatment of fibrosis, but the mechanisms are not totally understood (Nguyen et al., 2010; Park et al., 2019)**.** It might be beneficial if TGF-β inhibitors with proven effects are used with other metabolic regulators of fibrosis to achieve better treatment outcomes. A combined use of immune modulators or some existing glycolytic inhibitors together with off-market anti-fibrotic medications could proffer better treatment options for cardiac fibrosis.

Pharmacological interventions have been shown to improve cardiac function through inhibition of FA oxidation and improved glucose oxidation (Lopaschuk et al., 1988; Lopaschuk et al., 2010)**,** suggesting that metabolic rewiring may serve as an essential strategy**.** For instance, a recent study showed that deletion of a key enzyme in the FA oxidation pathway, CPT1b, not only reduced cardiac fibrosis but also stimulated cardiomyocyte proliferation post ischemia–reperfusion injury (Li et al., 2023b). Thus, it is possible, in principle, to pharmacologically block the activity of the CPT1b and open the door for the development of an inhibitor that can be used to effectively control the activity of the enzyme. This finding highlights the potential of metabolic reprogramming of other key enzymes and/or metabolites in the development of an effective anti-fibrotic therapy that may eventually be used in humans. Along these lines, we have recently reported that gene modulation and alteration make a substantial difference in the reaction of cells to the presence of pathological conditions or insults to the cells. We have shown that the deletion of delta-like 1 homolog (Dlk1) accelerates fibroblast–myofibroblast differentiation and that Dlk1-null mice have marked myocardial fibrosis (Rodriguez et al., 2019). It is interesting to know that Dlk1-null mice also exhibit accelerated adiposity (Moon et al., 2002), likely via increased preadipocyte replication/adipogenesis (Mortensen et al., 2012). Increased visceral adiposity contributes to fibroblast activation and cardiac fibrosis (Sawaki et al., 2018).

Further research would be important to understand the role of various gene alterations or manipulations on immune cell metabolism and changes that this metabolism could cause (Hinz et al., 2001; Van Den Borne et al., 2010; Hecker et al., 2011)**.** Although the mechanisms of myofibroblast deactivation/dedifferentiation are being investigated, many details still remain unknown (Fortier et al., 2021)**.** Efforts should be invested in strategies based on the manipulation of immune–metabolic pathways governing the function and phenotyping changes in myofibroblasts. As discussed earlier, macrophages can aid in the process of myocardial healing and fibrosis and may acquire multiple roles at different stages during wound resolution (Haider et al., 2019). In this regard, it is conceivable to assume that macrophages can unexpectedly participate in the modulation of myofibroblast dedifferentiation and myofibroblast removal/apoptosis or can undergo conversion into fibroblast-like cells, boosting interstitial collagen production to optimize healing (Haider et al., 2019)**.** Furthermore, therapies targeting receptor tyrosine kinase, such as anlotinib, have been shown to extract some anti-fibrotic effects in endometrial cancer and non-small cell lung cancer and to inhibit glycolysis in myofibroblasts to reverse pulmonary fibrosis (Chen et al., 2021b)**.** It will be interesting to know if it also confers the same benefits in cardiac fibrosis. Our rapidly expanding knowledge of CRISPR/Cas9 and RNA technologies in targeting specific mediators of myofibroblast dedifferentiation should possibly bring forward novel critical anti-fibrotic approaches (Figure 4).

## 6 Conclusion

Metabolic alterations in the myocardium associated with maladaptive hypertrophy and myocardial fibrosis induce a prolonged inflammatory response and energetic imbalance, which perturb cellular function and stimulate fibrotic events. An increasing number of studies have found that altered glycolysis, amino acid, and lipid metabolism in cardiac fibroblasts and immune cells induce an inflammatory environment and an extensive interdependent signaling contributes to the deposition of ECM during fibrosis. Simultaneously, these cellular and functional changes alter cellular metabolism, setting up a positive feedback chain of events that drive fibrosis. Hence, therapies targeting metabolic pathways in the immune cells or cardiac fibroblasts represent a very promising area of research for the treatment of cardiac fibrosis. However, the complexity of the immuno-metabolic networks in myocardial fibrosis creates challenges to therapeutic translation. Nevertheless, the similarities in metabolic derangements in glycolysis and β-oxidation across multiple fibrotic tissues should provide opportunities to target key metabolic pathways in cardiac fibrosis. Fibroblasts and macrophages are the primary cell types that mediate collagen internalization and degradation; therefore, reversing metabolic alterations must target both cells to effectively reduce fibrosis. Although the field of immunometabolism is burgeoning every day, there remain knowledge gaps about the contributions of *in vivo* immunometabolism directly within the myocardium. Moreover, much of our current insights into pathological myocardial remodeling originate from studies of myocardial ischemia, and there is a much larger knowledge gap between immune cell metabolism and nonischemic, cardiometabolic heart disease. While some anti-fibrotic drugs targeting metabolic dysregulation have shown promising results, more rigorous clinical studies are needed to test their therapeutic efficacy and adverse effects. In this respect, drug repurposing strategies in parallel with systematic, large-scale drug screening should be highly considered (Zhao et al., 2019). Moreover, further research is still required to elucidate how the interplay of inflammation and metabolic rewiring promotes fibrosis and what is the difference in the immunometabolism during physiological tissue repair and pathophysiological fibrotic response.
